# Associations between generative AI use frequency, technology acceptance, attitudes toward AI, and reported learning preference patterns among students in physical education classes

**DOI:** 10.3389/fspor.2026.1806871

**Published:** 2026-06-15

**Authors:** Stefan Alecu, Gheorghe Adrian Onea

**Affiliations:** Department of Physical Education and Special Motricity, Transilvania University, Brașov, Romania

**Keywords:** attitudes toward AI, cross-sectional survey, generative artificial intelligence, learning preferences, physical education, technology acceptance model

## Abstract

**Introduction:**

Physical education combines embodied practice with cognitive learning, yet generative artificial intelligence is increasingly used as an academic support tool. This cross-sectional study investigated associations between self-reported generative AI use frequency, reported VARK learning preference patterns, attitudes toward AI, and Technology Acceptance Model beliefs among undergraduate students in PE classes.

**Methods:**

A cross-sectional survey was conducted with 1,084 full-time PE undergraduates (age 19–28 years). Participants completed PE-adapted instruments assessing reported learning preferences patterns using the VARK framework, technology acceptance beliefs based on the Technology Acceptance Model, attitudes toward generative AI, and self-reported generative AI use frequency. The TAM and VARK instruments were adapted to PE-relevant academic and movement-related tasks. Data were analyzed using descriptive statistics, reliability testing, ANOVA, and multiple regression models.

**Results:**

Students reported moderate acceptance of generative AI and positive attitudes toward its academic use. Kinesthetic learning remained the dominant preference, consistent with the movement-based nature of PE. However, higher self-reported AI usage frequency was associated with higher visual and reading-writing scores and lower kinesthetic scores (*β* = .34–.40; *β* = −.39, *p* < .001). Significant differences emerged across learning-style groups for all TAM and AI attitude dimensions (*p* < .001, *η*^2^ = .086–.142). Regression analysis showed that perceived usefulness, ease of use, and positive attitudes toward AI were the strongest predictors of AI usage, explaining 48% of variance. Frequent AI engagement was associated with greater concentration in in reported VARK score patterns (*β* = −.62, *p* < .001).

**Conclusions:**

In this cross-sectional sample, self-reported generative AI use was associated with differences in reported learning preference patterns and with stronger technology acceptance beliefs among higher education physical education students. While kinesthetic scores remained highest overall, more frequent AI use was associated with higher visual and reading-writing scores and lower kinesthetic scores. These findings are associative, not causal, and do not show changes in stable learning preference patterns. Future research should examine whether pedagogically grounded AI integration can support diverse learners, particularly students who report stronger kinesthetic preferences.

## Introduction

Physical education (PE) occupies a distinctive position within school curricula because it combines cognitive learning with embodied, movement-based experiences. Through regular participation in PE, students are expected to improve physical fitness, motor competence, and confidence, while also developing AI-related perceptions and positive attitudes toward lifelong physical activity. However, these outcomes depend strongly on instructional quality and the learning environment students experience. Student motivation remains one of the most important determinants of engagement and learning in PE. Motivated students participate more actively, persist through difficulty, and transfer positive experiences into physical activity beyond school. Self-Determination Theory (SDT) provides a useful theoretical background for understanding how educational environments may support autonomy, competence, and relatedness, which are linked to student engagement in physical education (1, 2). However, the present study did not directly measure SDT constructs or AI-related perceptions. Accordingly, SDT is used here only as a broad interpretive framework, while the empirical analyses focus specifically on attitudes toward generative AI, technology acceptance beliefs, self-reported AI use frequency, and reported learning preference patterns. At the same time, learners differ in how they engage with movement-based instruction. Some students may report preferences for particular instructional formats, such as visual modelling, verbal explanation, or repeated physical practice. However, contemporary educational research has questioned strong learning-styles claims, especially the assumption that such preferences represent stable traits or that instruction is more effective when rigidly matched to a preferred modality. For this reason, the present study uses VARK scores as indicators of reported preference patterns within a specific context, rather than as evidence of fixed reported learning preference patterns. Recent pedagogical work suggests that instructional variety, rather than rigid learning-style matching, can enhance perceived competence and autonomy, supporting motivation indirectly (3, 4). Digital technologies increasingly shape PE teaching through blended and hybrid models. These approaches combine in-person instruction with digital resources and have been linked to improved engagement and autonomy when applied carefully (5). Within this transformation, artificial intelligence (AI) has emerged as a major innovation in physical education.

However, little is known about how generative AI use is associated with students' attitudes toward AI, technology acceptance beliefs, and reported learning preferences in authentic PE contexts. AI systems can process large volumes of student performance data, support assessment, provide immediate feedback, and assist teachers in adapting tasks to learner needs (6). Sport science literature shows that wearable sensors and AI-based monitoring already support performance optimization and injury prevention (7, 8). Video-based deep learning tools further extend feedback possibilities in movement learning (9). As traditional analytical methods struggle with complex datasets, AI provides new opportunities for real-time decision support in sport and education contexts (10, 11). In addition to these applied developments, foundational scholarship has highlighted the broader conceptual role of AI in sport. Dhar (12) argued that AI can enhance sports contexts by analyzing large-scale performance data, supporting decision-making, and modelling complex behavioural patterns. This perspective provides an important theoretical basis for extending AI tools into movement-based educational environments such as PE, where algorithmic insights may strengthen coaching feedback, adaptive instruction, and learner-centred support.

Generative AI expands these applications by producing adaptive learning materials, personalized feedback, and differentiated guidance in real time. This aligns closely with autonomy-supportive PE instruction because students can access content through multiple formats and progress at an individual pace. Explainable AI frameworks also highlight the importance of transparency and responsible implementation when AI is introduced into learning environments (13, 14). Recent educational research further suggests that generative AI can directly support learner motivation and cognitive engagement. Monzon and Hays (15) emphasize that GenAI tools can foster autonomy, enhance AI-related perceptions, and strengthen knowledge retention through personalized feedback, flipped learning models, and interactive retrieval-based strategies (16, 17). Empirical evidence also indicates that deep learning-based AI models can predict both academic success and student engagement, linking engagement analytics with performance outcomes in physical higher education students (18). Such findings demonstrate the growing potential of AI not only to personalize instruction but also to anticipate motivational and engagement patterns crucial for learning in PE.

Evidence from AI-supported physical activity interventions suggests positive motivational effects. Meta-analytic findings indicate that exergames and interactive AI technologies produce moderate improvements in learning outcomes and student interest (19, 20). Deep-learning systems have also been developed for personalized fitness evaluation and exercise prescription, increasing perceived relevance and engagement in PE contexts (21). Broader sport AI research confirms that machine learning supports coaching, performance prediction, talent identification, and training optimization (22–24). Recent narrative reviews emphasize that AI is transforming sport into a data-rich domain, integrating biomechanics, health monitoring, and adaptive coaching support (25). These advances create strong potential for PE teaching, yet they also raise challenges involving privacy, ethics, and educational adoption (26, 27). Despite these developments, research on generative AI in physical education remains limited. Most studies focus on technical feasibility rather than examining how generative AI is associated with students' attitudes toward AI, technology acceptance beliefs, and reported learning preference patterns in authentic PE settings (6). This gap highlights the need for research that connects AI innovation with learner diversity and technology acceptance in movement-based education.

In the present study, these variables were combined within an association-based conceptual framework. Technology acceptance beliefs, specifically perceived usefulness, perceived ease of use, and attitude toward using AI, were included because prior research suggests that they are closely related to willingness to engage with digital tools in educational settings. Attitudes toward generative AI, including interest, behavioral intention, and perceived safety, were included as complementary perception-based variables that may also be associated with reported AI engagement. Reported VARK modality scores were included not as fixed reported learning preference patterns, but as context-dependent indicators of how students report preferring to engage with learning tasks. Within this framework, the study examined whether reported AI engagement co-occurred with particular reported preference patterns, and whether these associations were also related to technology acceptance beliefs and general attitudes toward AI.

The aim of this study was to examine cross-sectional associations between self-reported generative AI use frequency, technology acceptance beliefs, attitudes toward AI, and reported learning preference patterns (VARK) among undergraduate students in PE classes. More specifically, the study examined whether reported AI engagement was associated with technology acceptance beliefs and attitudes toward AI, and whether these variables were also associated with reported VARK modality patterns within the PE learning context. We formulated the following association-based hypotheses:

H1: Higher self-reported generative AI use frequency will be associated with higher visual and reading/writing preference scores and lower kinesthetic preference scores.
H2: More positive attitudes toward AI will be associated with higher perceived usefulness and perceived ease of use.
H3: Higher TAM scores will be associated with more frequent self-reported generative AI use; exploratory analyses will examine whether self-reported AI use frequency is associated with learning profile concentration.

## Materials and methods

### Participants

The study sample consisted of 1,084 undergraduate students enrolled in physical education programs across various academic years. Among them, 468 were female and 616 were male, aged between 19 and 28 years (M = 21.03, SD = 1.89). All participants were full-time students with access to digital learning tools and had used at least one educational platform in the past six months. Recruitment occurred during compulsory coursework sessions, where faculty members presented an invitation to participate in the study. Participation was entirely voluntary, and all individuals provided informed consent prior to data collection. Participants retained the right to withdraw at any stage. Data were collected electronically, and only fully completed responses were retained for analysis. Demographic variables were recorded to characterize the sample, and no personally identifiable data were collected at any stage.

All study procedures were approved by the Institutional Review Board of the Faculty of Physical education and mountain sports under the document no. 183/14.09.2025, and were conducted in accordance with the Declaration of Helsinki for human subjects research. The study was classified as minimal risk due to the non-invasive nature of the data collection methods.

### Study design

This research employed a cross-sectional design aimed at examining associations involving generative artificial intelligence (AI) use in academic activities among physical education students. The analysis focused on three core domains: (1) reported learning preference patterns as assessed by the VARK questionnaire, (2) attitudes toward AI-based tools, and (3) technology acceptance, operationalized through the Technology Acceptance Model (TAM). These domains were included for complementary conceptual reasons. VARK scores were treated as context-dependent indicators of reported modality preferences in learning tasks. TAM variables were included to capture core technology acceptance beliefs expected to relate to reported AI engagement. The attitudes toward generative AI scale was included to capture broader affective and evaluative orientations toward AI, beyond perceived usefulness and ease of use. Accordingly, the study was designed to examine how reported preference patterns, acceptance beliefs, and AI-related attitudes were associated with self-reported academic AI engagement within a single cross-sectional framework. All participants completed the same set of instruments in a single data collection session, with the administration order standardized to control for response-order effects. Participants completed the survey in a supervised academic environment using personal internet-enabled devices. Clear procedural instructions were provided to minimize external disruptions. The estimated time to complete all instruments was approximately 20 min. The online platform used ensured data privacy and security, allowing only valid responses and filtering out duplicates. All participants received a standardized introduction outlining the study purpose and procedures. The study design enabled both subgroup comparisons and relational analyses across the targeted constructs. The data supported hypothesis testing regarding associations among self-reported generative AI use, attitudes toward AI, technology acceptance beliefs, and reported learning preference patterns within the field of physical education.

### Instruments

#### VARK learning preference inventory v.8.02 (PE adapted version)

The study used a physical-education-adapted version of the VARK inventory to assess students' reported preference patterns across the Visual, Auditory, Reading/Writing, and Kinesthetic modalities. The adaptation preserved the original structure of the instrument, while item wording was aligned with tasks commonly encountered in university-level physical education, such as learning movement sequences, analyzing technique, and integrating digital training tools. This improved contextual relevance for the target population. The response format allowed multiple selections per item, and each response was coded into the corresponding modality to generate modality score patterns for each participant. In line with current criticism of learning-styles frameworks, these scores were not treated as fixed cognitive traits or as evidence that students learn best only when instruction matches one modality. Instead, they were used as context-dependent, self-reported indicators of preferred ways of engaging with learning tasks in the PE setting. Although the adaptation was designed to improve contextual relevance, the present study did not test the factor structure or broader construct validity of the adapted version in the PE context.

#### Technology acceptance model (TAM) (PE adapted version)

Perceptions of generative AI use were assessed with a physical-education-adapted version of the Technology Acceptance Model. The instrument retained the original two-factor structure, measuring perceived usefulness and perceived ease of use, and used the same five-point Likert response format. Items were adapted to reflect academic and practical learning activities specific to physical education, such as understanding movement principles, planning training tasks and preparing assessments with AI support. AI usage frequency was measured using a self-reported 5-point scale (1 = very rarely, 5 = very frequently) assessing how often students used generative AI tools for academic purposes. This variable was treated as a self-reported indicator of perceived academic AI engagement rather than an objective behavioral measure. It did not assess type of use, specific purpose, duration of use, platform-specific behavior, or objectively verified interaction patterns. The adaptation was intended to preserve the original constructs, but full construct validation was not performed in the present sample. The instrument was translated into Romanian using standard translation and back-translation procedures to ensure semantic accuracy. This approach supports contextual relevance, although further validation would strengthen measurement rigor by preserving construct integrity and enhancing measurement precision in the target population. Although the adaptation was intended to preserve the original constructs while improving contextual alignment, full psychometric validation of the PE-adapted version, including factor-analytic testing, was not performed in the present sample.

The Technology Acceptance Model instrument was adapted to the physical education context by wording items around PE-relevant academic and professional tasks. Specifically, perceived usefulness, perceived ease of use, and attitude toward using AI were framed in relation to activities such as understanding biomechanical concepts, analysing movement skills, preparing training plans, receiving feedback on assignments, and supporting theoretical learning in PE-related courses. This adaptation was intended to improve contextual relevance while preserving the conceptual structure of the original TAM dimensions. Internal consistency reliability was assessed using Cronbach's alpha. However, high alpha values were interpreted as evidence of internal consistency in this sample, not as complete evidence of construct validity.

#### Attitudes toward generative AI scale (PE adapted version)

Students' attitudes toward generative AI were measured using a scale adapted for physical education. The scale included three dimensions, interest in using AI for academic purposes, behavioral intention to adopt AI tools and perceived safety when interacting with generative AI systems. The adaptation kept the original dimensional structure, but items were rewritten to reflect the learning context of physical education, including movement analysis tasks, study routines and digital learning materials. This ensured that the instrument captured attitudinal and engagement-related perceptions relevant to students' actual experiences with AI in this field. The five-point Likert response structure was preserved to maintain comparability with existing studies. The adaptation improved contextual relevance while retaining the validity of the original constructs. In this study, attitudes toward generative AI (e.g., interest, behavioral intention, perceived safety) are treated as perception-based indicators of attitudes and engagement-related perceptions, rather than as direct measures of AI-related perceptions within a formal SDT framework. Learning profile clarity was operationalized as the dispersion of scores across the four VARK modalities, calculated as the standard deviation of the four modality scores for each participant. Lower values indicate greater concentration of preferences within a dominant modality, reflecting a clearer learning profile, whereas higher values indicate more distributed (multimodal) preference patterns. Thus, the adapted scale should be interpreted as a context-sensitive measure of reported AI-related perceptions in PE, although additional validation would be needed to confirm its construct structure in this population.

### Procedure

Data collection was conducted over a 10-week period during the academic semester, between October and December 2025, to ensure adequate recruitment and representativeness across multiple cohorts of physical education students. This extended timeframe allowed the research team to approach students enrolled in compulsory coursework sessions across different academic years and to achieve the large final sample size of 1,084 participants. A total of approximately 1,200 undergraduate students enrolled in physical education programs were invited to participate in the study during compulsory coursework sessions. Of these, 1,112 students agreed to take part and accessed the online survey. After screening for completeness, 1,084 fully completed responses were retained for the final analysis. Incomplete submissions and duplicate entries were excluded, resulting in a final completion rate among those who accessed the survey was 97.5%; retention rate from invited students was 90.3% based on the number of students who initiated participation. Survey administration was scheduled in supervised university settings, ensuring consistent procedures and minimizing disruptions. The multi-week recruitment window also reduced selection bias by enabling participation from students attending classes on different dates and supported the inclusion of both male and female students from diverse study years. This duration is consistent with large-scale cross-sectional survey research in higher education contexts and provided sufficient time to obtain complete responses while maintaining standardized data collection conditions. Generative AI use was measured through self-reported frequency of use for academic purposes. Students were asked to report how often they used generative AI tools to support PE-related learning activities, including summarising information, explaining theoretical or biomechanical concepts, supporting academic research, drafting or revising assignments, and obtaining feedback on learning materials. The study did not collect platform log data, exact usage duration, prompt histories, or independent verification of the quality of AI-supported learning activities.

### Inclusion and exclusion criteria

Participants were eligible for inclusion if they were full-time undergraduate students enrolled in physical education programs and had used at least one generative AI or digital learning tool within the previous six months. Students were required to have regular access to internet-enabled devices and to be able to complete the online survey independently. Responses were included in the final dataset only if all questionnaire items were fully completed. Surveys with missing data, incomplete submissions, or duplicate entries identified by the online platform were excluded from analysis.

### Data analysis

The analyses were guided by an association-based conceptual model rather than a causal path model. In this framework, reported AI engagement was treated as the central behavioral-perceptual outcome, technology acceptance beliefs and attitudes toward AI were treated as theoretically relevant correlates of reported AI engagement, and VARK modality scores were treated as contextual preference indicators that may be associated with both AI engagement and AI-related perceptions. The regression analyses were therefore used to estimate the strength of associations among these constructs, not to test a directional causal sequence. Data analysis was computed using SPSS v.26, proceeded in two phases. The first involved descriptive statistics to summarize the distribution of the primary variables. The second phase addressed the relationships between reported learning preference patterns (VARK), attitudes toward AI, and technology acceptance (TAM). VARK responses were categorized into dominant reported learning preference patterns for each participant. Mean scores were computed for both TAM subscales and the attitude scale. Internal consistency was assessed using Cronbach's alpha coefficients. Group comparisons (by gender or learning style) were conducted using standard inferential statistics, including *t*-tests and ANOVA. In cases of tied highest modality scores, participants were classified as multimodal and excluded from dominant-profile ANOVA. Furthermore, regression models were developed to estimate the associations of TAM and AI attitudes with reported learning preference patterns and generative AI usage patterns. This approach is consistent with recent research examining students' perceptions of generative AI tools, which emphasizes attitudes, perceived usefulness, and behavioral intention rather than direct measurement of formal motivational constructs (28). AI usage frequency was measured using a self-reported Likert-type scale assessing how often students reported engaging with generative AI tools for academic purposes, from low to high frequency. Accordingly, the regression models should be interpreted as estimating associations with reported AI engagement rather than objective adoption behavior or verified usage patterns in academic contexts. Normality, multicollinearity, homogeneity were checked. Learning profile clarity was operationalized as the dispersion of scores across the four VARK modalities, with lower dispersion indicating greater concentration of scores within one or fewer dominant modalities. Although some predictors are conceptually related (e.g., attitude toward use and behavioral intention), all variables were retained to examine their unique contributions within the regression models. Regression models were used to estimate associations under different analytical specifications and should not be interpreted as directional or causal models.

For the purpose of subgroup comparisons, each participant was assigned a dominant VARK preference profile based on their highest modality score on the VARK inventory. The dominant modality was defined as the category, Visual, Auditory, Reading/Writing, or Kinesthetic, with the greatest number of selected responses. Participants were then grouped accordingly to examine differences in technology acceptance and attitudes toward generative AI across reported VARK categories. These categories were used as analytical groupings derived from self-reported modality scores, not as evidence of stable reported learning preference patterns or functionally determinative learner types. Because all variables were self-reported and the instruments were adapted for the PE context, the analyses should be interpreted as examining associations among reported constructs rather than causal effects or changes over time.

## Results

The analyses examined relationships between students' learning preferences, attitudes toward generative AI and acceptance of AI tools in physical education. Descriptive statistics were first calculated for all subscales of the PE-adapted VARK, TAM and Attitudes Toward Generative AI instruments.

The descriptive results presented in Table 1 show clear and interpretable patterns across all three PE-adapted instruments. Scores for perceived usefulness and ease of use (TAM) cluster around the midpoint of the scale, indicating that students see generative AI as moderately supportive and accessible in physical education learning tasks. Attitudes toward AI display a similar pattern, with interest, intention and perceived safety producing stable means and low distributional distortion. These values suggest that students hold generally positive but measured views toward academic use of AI.

**Table 1:** Descriptive statistics for the three PE-adapted instruments.

Subscale	X	SD	CV%	95% CI		Skewness	Kurtosis	*p*	d
				LL	UL				
TAM_PU	3.08	0.81	26.35	3.03	3.13	−0.08	−0.28	0.287	0.066
TAM_PEOU	3.00	0.85	28.17	2.95	3.05	−0.07	−0.41	0.807	0.006
TAM_ATU	3.18	0.76	24.00	3.13	3.22	0.02	−0.25	0.476	0.040
AI_interest	3.47	0.96	27.63	3.41	3.53	−0.05	−0.54	0.598	0.032
AI_behavioral_intention	3.41	1.03	30.20	3.35	3.47	−0.06	−0.60	0.759	−0.019
AI_safety	3.40	0.87	25.59	3.35	3.45	−0.04	−0.44	0.911	−0.004
VARK_V	6.01	3.13	52.08	5.82	6.20	0.14	−0.32	0.622	−0.021
VARK_A	6.03	3.17	52.56	5.84	6.22	0.16	−0.30	0.710	−0.017
VARK_R	5.99	3.17	52.95	5.80	6.18	0.15	−0.32	0.836	−0.013
VARK_K	10.03	3.21	32.04	9.84	10.22	−0.10	−0.47	0.265	−0.069

X, mean; SD, standard deviation; CV, coefficient of variation; 95% CI, 95% confidence interval; *p*, statistical significance threshold; d, Cohen's d.

Learning preferences show a distinct profile. Kinesthetic scores are highest, which aligns with the practical and movement-centered nature of physical education. Visual, auditory and reading-writing preferences are moderate and display wider variability, suggesting that students incorporate multiple modes when interacting with digital or AI-based content. The coefficient of variation indicates that the greatest dispersion appears in the VARK subscales, which supports the notion that learning preferences in physical education are diverse and sensitive to instructional format.

Across all variables, skewness and kurtosis remain close to zero, indicating balanced distributions suitable for parametric analyses. This distributional stability strengthens the reliability of subsequent correlational and inferential testing. The absence of significant gender differences (all *p* values > .05, Cohen's d near zero) confirms that the constructs operate similarly across male and female students, allowing the full sample to be treated as a unified analytical group.

Taken together, the descriptive data reveal a student population with moderately positive attitudes toward AI, stable perceptions of its usefulness and ease of use, and heterogeneous yet coherent learning preferences. These patterns provide an empirical basis for examining the study's hypotheses on how AI attitudes and TAM beliefs relate to digital learning tendencies and VARK profiles.

Because the instruments were adapted to the PE context, future research should further examine their factorial structure, measurement invariance, convergent validity, and predictive validity across different PE populations. Both TAM dimensions reach very high values (*α* ≈ .96), indicating strong internal consistency as shown in Table 2; however, such high values may also suggest item redundancy rather than broader construct coverage in the physical education context. The two-item Attitude Toward Using subscale also shows good reliability (*α* = .87), which is notable given the typical difficulty of achieving strong internal consistency with very short scales.

**Table 2:** Cronbach's alpha (*α*) for all PE-adapted subscales.

Subscale	*α*
TAM_PU	0.961
TAM_PEOU	0.964
TAM_ATU	0.875
AI_interest	0.932
AI_behavioral_intention	0.934
AI_safety	0.912

*α*, Cronbach's *α*.

The Attitudes Toward Generative AI dimensions display similarly strong reliability, with coefficients above .91 across interest, intention and perceived safety. These results suggest that the adapted items showed internal consistency coherent and well-defined constructs related to students' attitudinal and engagement-related orientations toward AI use. Although Cronbach's alpha values for TAM_PU and TAM_PEOU were very high (*α* > .96), this may indicate item redundancy rather than increased construct breadth, which should be considered when interpreting these scales.

Overall, the reliability evidence suggests that the adapted scales showed strong internal consistency in the present sample. However, these results should not be interpreted as full evidence of construct validity, because internal consistency alone does not confirm factor structure, measurement equivalence, or broader validity in the PE context.

The ANOVA results presented in Table 3 revealed statistically significant differences across learning-style groups for all TAM and Attitudes Toward AI subscales. F statistics ranged from 33.71 to 59.42, all with *p* values far below the .001 threshold. Effect sizes (*η*^2^ between .086 and .142) indicate moderate practical significance, suggesting that dominant VARK preference profile were meaningfully associated with how students perceive and interact with generative AI in physical education.

**Table 3:** Statistical results for ANOVA results by learning style.

Variable	F	*p*	*η* ^2^
TAM_PU	54.998	*p* < .001	0.133
TAM_PEOU	51.388	*p* < .001	0.125
TAM_ATU	59.425	*p* < .001	0.142
AI_interest	47.331	*p* < .001	0.116
AI_behavioral_intention	45.942	*p* < .001	0.113
AI_safety	33.710	*p* < .001	0.086

F, one-way ANOVA test; *p*, statistical significance threshold; *η*^2^ (eta squared, effect size.

Students with dominant visual and reading-writing preferences consistently reported higher perceived usefulness, ease of use and positive attitudes toward AI-based learning tools. In contrast, those with a predominantly kinesthetic profile tended to score lower on these dimensions, reflecting less alignment between hands-on learning preferences and digital or AI-mediated instructional formats. These findings show that AI perceptions differed across reported VARK profile groups.

These findings are consistent with H1, showing that students with different dominant reported learning preference profiles also differed in their perceptions of generative AI and technology acceptance.

The gender comparisons presented in Table 4 showed that male and female students scored almost identically across all subscales of the PE-adapted TAM, Attitudes Toward Generative AI and VARK instruments. Mean differences were minimal, and the associated t values were small, indicating an absence of meaningful statistical variation between genders. For all measures, standard deviations were nearly identical as well, suggesting similar response dispersion across male and female participants.

**Table 4:** Gender-based descriptive statistics and t-values for PE-adapted TAM, attitudes toward AI and VARK subscales.

Subscale	Gender	X	SD	t
TAM_PU	F	3.10	0.81	1.07
	M	3.06	0.81	
TAM_PEOU	F	3.00	0.85	0.24
	M	3.00	0.84	
TAM_ATU	F	3.19	0.76	0.71
	M	3.17	0.76	
AI_interest	F	3.48	0.95	0.53
	M	3.46	0.96	
AI_behavioral_intention	F	3.42	1.02	0.31
	M	3.41	1.03	
AI_safety	F	3.40	0.87	0.11
	M	3.40	0.87	
VARK_V	F	6.00	3.11	−0.50
	M	6.02	3.14	
VARK_A	F	6.04	3.18	−0.38
	M	6.02	3.17	
VARK_R	F	6.00	3.16	−0.21
	M	5.99	3.18	
VARK_K	F	10.10	3.20	1.12
	M	9.98	3.22	

X, mean; t, independent samples *t*-test.

Overall, the pattern of results demonstrates that gender does not influence students' perceptions of AI usefulness, ease of use or attitudes toward generative AI. Likewise, learning preferences measured through VARK did not differ by gender. This uniformity supports the interpretation that both male and female students engage with AI-based learning tools in comparable ways and exhibit similar learning tendencies within physical education programs. The lack of gender effects also indicates that subsequent analyses can be conducted on the full sample without the need for gender-specific modeling or statistical control.

The regression results presented in Table 5 show consistent associations across all four VARK dimensions. Self-reported AI usage frequency showed the strongest positive association with Visual and Reading/Writing scores and was negatively associated with Kinesthetic scores. These associations were substantial in magnitude (*β* = .34–.40 for Visual and Reading/Writing, *β* = −.39 for Kinesthetic, all *p* < .001), indicating that more frequent self-reported AI engagement co-occurred with higher scores on the visual and reading/writing dimensions and lower scores on the kinesthetic dimension. This pattern aligns with the expectations of Hypothesis 1 at the associative level.

**Table 5:** Multiple regression models estimating associations with VARK scores.

Predictor	Visual		Auditory		Read/Write		Kinesthetic	
	*β*	*p*	*β*	*p*	*β*	*p*	*β*	*p*
AI_use_freq	.38	<.001	.34	<.001	.40	<.001	−.39	<.001
TAM_PU	.09	.028	.08	.048	.08	.042	−.09	.009
TAM_PEOU	.06	.164	.05	.284	.05	.215	−.06	.121
TAM_ATU	.11	.014	.10	.032	.11	.020	−.11	.001
AI_interest	.05	.211	.04	.308	.05	.196	−.05	.119
AI_behavioral_intention	.05	.134	.05	.196	.04	.201	−.04	.225
AI_safety	.03	.405	.02	.542	.02	.568	−.02	.432

R^2^ = .26 (26% of the variance) for Visual; .22 (22% of the variance) for Auditory; .29 (29% of the variance) for Reading/Writing; .31 (31% of the variance) for Kinesthetic.

*β*, standardized beta coefficient.

TAM variables contributed additional explanatory power. Perceived usefulness and attitude toward using AI were significant predictors across all VARK dimensions. Higher usefulness and more positive attitudes were associated with stronger Visual and Reading/Writing preferences and lower Kinesthetic orientation. These findings suggest that students who perceive AI tools as beneficial and relevant also report preference patterns that align more closely with AI-supported formats, such as visual explanations, textual prompts and structured feedback. However, because the data are cross-sectional, these results do not indicate whether self-reported AI use is linked to reported preference patterns because AI use is greater among students with more visual or text-based preferences, because such preferences are associated with more frequent AI use, or because both reflect broader tendencies in academic engagement.

Attitudes toward AI (interest, intention and safety) displayed weaker and mostly nonsignificant effects, indicating that cognitive evaluations of AI as captured by TAM, were more strongly associated with reported VARK scores than emotional or motivational dispositions in the present sample.

Overall, the model explains between 22% and 31% of the variance in VARK dimensions, which indicates moderate explanatory value for self-reported VARK score patterns. The pattern of results supports the view that generative AI engagement and positive technology perceptions are associated with more digitally oriented reported VARK patterns and lower kinesthetic scores in physical education contexts.

The regression model predicting generative AI usage presented in Table 6 was significant and accounted for 48% of the variance in usage frequency. Higher perceived usefulness, ease of use and positive attitudes toward using AI were the strongest predictors of increased AI engagement. Behavioral intention and perceived safety also contributed significantly, while AI interest showed a marginal effect. These results indicate that both TAM dimensions and attitudinal factors play meaningful roles in shaping how frequently students use generative AI in academic physical education contexts.

**Table 6:** Regression model estimating associations with self-reported generative AI usage frequency.

Predictor	B	SE B	*β*	t	*p*
Constant	−0.99	0.12	—	8.08	<.001
TAM_PU	0.29	0.04	.28	6.92	<.001
TAM_PEOU	0.25	0.04	.25	6.08	<.001
TAM_ATU	0.30	0.04	.27	6.68	<.001
AI_interest	0.08	0.04	.08	1.92	.056
AI_behavioral_intention	0.14	0.04	.13	3.28	.001
AI_safety	0.09	0.04	.08	2.12	.034

R^2^ = .48 (48% of the variance).

B, unstandardized regression coefficient; SE B, standard error of B; *β*, standardized beta coefficient; t, t-statistic for each predictor.

The regression model presented in Table 7 indicates that AI usage frequency was the dominant predictor of VARK profile concentration, with a large negative standardized beta (*β* = −.62, *p* < .001). In the present operationalization, lower dispersion values indicate greater concentration of responses within one or fewer dominant VARK modalities. Accordingly, higher AI use was associated with more concentrated reported VARK score patterns. Importantly, none of the TAM components nor the attitudinal variables significantly predicted this index once AI use was included in the model. Because this variable is derived from self-reported VARK scores in a cross-sectional design, it should not be interpreted as evidence of more stable, better, or developmentally clearer reported learning preference patterns; rather, it reflects concentration in reported modality scores. For this reason, this analysis should be treated as exploratory. Multiple regression analyses were conducted to examine the predictive relationships between generative AI usage frequency, technology acceptance variables (TAM), attitudes toward AI, and learning preference outcomes (VARK dimensions). All regression models were specified using the enter method, whereby all predictors were entered simultaneously to estimate their unique contribution to each dependent variable. All analyses were performed using SPSS version 26, with statistical significance set at *p* < .05. Prior to model estimation, key statistical assumptions were assessed. Normality of residuals and linearity of relationships were examined through standardized residual plots. Homoscedasticity was evaluated by inspecting the distribution of residuals across predicted values. Multicollinearity was assessed using variance inflation factors (VIF) and tolerance values. All predictors demonstrated acceptable collinearity levels (VIF < 5), indicating that intercorrelations among independent variables did not bias regression estimates.

**Table 7:** Regression predicting learning profile clarity from AI Use, TAM and AI attitudes.

Predictor	B	SE B	*β* (std.)	t	*p*
Constant	8.9105	0.4509	—	19.75	<.001
AI_use_freq	−1.6126	0.1095	−.62	14.73	<.001
TAM_PU	−0.1570	0.1514	−.07	1.04	.300
TAM_PEOU	−0.0120	0.1510	−.01	0.08	.937
TAM_ATU	−0.0529	0.1631	−.03	0.33	.746
AI_interest	0.0933	0.1503	.04	0.62	.535
AI_behavioral_intention	−0.0715	0.1526	−.03	0.47	.639
AI_safety	−0.0781	0.1555	−.03	0.50	.616

R^2^ = .309 (31% of the variance).

Model fit was evaluated using the coefficient of determination (R^2^), reflecting the proportion of variance explained by each regression equation.

## Discussion

This study identified cross-sectional associations among self-reported generative AI use, attitudes toward AI, technology acceptance beliefs, and reported learning preference patterns within a single conceptual framework. In that framework, technology acceptance beliefs and broader AI attitudes were treated as perception-based correlates of reported AI engagement, while reported VARK modality scores were treated as contextual indicators of how students report engaging with learning tasks. The results therefore speak to how these constructs co-occur within a physical education context, rather than to a single causal chain among them in higher education physical education. The results showed moderate acceptance of AI tools and generally positive attitudes toward AI, alongside associations between self-reported AI use frequency and differences in reported VARK scores. These findings indicate co-occurring patterns within the sample rather than directional effects. In line with current educational psychology perspectives, VARK scores in the present study should be interpreted as context-dependent, self-reported learning preference patterns rather than stable cognitive styles. More specifically, they should not be understood as functionally determinative learner characteristics or as evidence that students learn more effectively when instruction is matched to a single preferred modality. Accordingly, the findings should be interpreted as associations among self-reported constructs, not as evidence that AI changes motivation, causes shifts in stable reported learning preference patterns, or produces developmental effects over time. In this study, self-reported AI use should be understood as reported or perceived engagement with generative AI for academic purposes, not as an objective behavioral record of how students actually used AI systems.

Because the study used a cross-sectional design and self-reported, PE-adapted instruments, the findings should be interpreted as associative rather than causal. The present study focuses on technology acceptance (TAM) and attitudes toward generative AI, self-reported AI use frequency, and reported learning preference patterns, rather than directly measuring motivation within a Self-Determination Theory framework. A further methodological consideration concerns the measurement of generative AI use. In this study, AI engagement was operationalised through self-reported frequency. Although this approach is appropriate for examining broad adoption patterns in a large cross-sectional sample, it does not capture the quality, duration, platform type, prompt complexity, or pedagogical appropriateness of actual AI use. For example, occasional high-quality use of AI for biomechanical explanation or training-plan development may differ substantially from frequent but superficial use for summarisation or assignment drafting. Therefore, the present findings should be interpreted as reflecting perceived and reported AI engagement rather than objectively verified AI-supported learning behaviour. Physical education depends on experiential learning. Students build competence through action, repetition, coordination, and sensory feedback. Motor learning research confirms that skill acquisition relies on practice conditions that integrate perception and movement in realistic contexts (29, 30, 42). The data are consistent with this foundation, as kinesthetic scores remained highest across the sample. At the same time, more frequent self-reported AI use co-occurred with stronger visual and reading-writing scores. This pattern may reflect an association between reported AI engagement and greater use or preference for screen-based, textual, visual, and feedback-oriented academic support formats However, the present design does not allow conclusions about whether AI use produced these patterns. Multimedia learning theory may offer one possible interpretive lens for these associations, but such interpretation remains speculative in the absence of longitudinal or experimental evidence (31). In PE, these associations may be relevant in contexts where students report using AI for technique explanations, biomechanics concepts, or tactical understanding. This pattern does not indicate that kinesthetic learning disappears or that AI use directly shifts students away from embodied forms of learning. Rather, it may reflect that students who report more frequent AI use also report learning preference patterns that are more compatible with text- and visual-based forms of cognitive scaffolding. Contemporary sport pedagogy stresses that digital tools can extend learning beyond the gym by supporting reflection, planning, and analysis (32).

A key contribution of our study is the strong association between technology acceptance beliefs and self-reported AI use frequency. Perceived usefulness, ease of use, and positive attitudes toward AI were associated with a large proportion of variance in AI usage frequency. This aligns with decades of evidence that usefulness remains the most consistent driver of educational technology adoption (33). In PE contexts, usefulness likely reflects practical benefits such as faster access to learning materials, improved understanding of movement tasks, and support for academic workload. Research on digital learning adoption in sport programs shows that students engage when technology offers direct performance or study advantages (43). Ease of use also matters. Generative AI systems lower barriers by providing conversational, immediate support. This matches findings that reduced effort increases sustained engagement with learning platforms (34). In physical education, where students balance theory with practice, tools that simplify cognitive demands become especially attractive.

The ANOVA results reveal that learning-style groups differ significantly in their AI perceptions. Visual and reading-writing learners report higher usefulness and stronger intentions to adopt AI, while kinesthetic learners show weaker alignment. This reflects an important pedagogical tension. Generative AI currently operates mainly through text and visual outputs. These formats naturally suit learners who already prefer symbolic information channels. Students with stronger kinesthetic preference scores may place greater emphasis on bodily engagement within PE learning tasks. Research in embodied cognition emphasizes that understanding in movement disciplines develops through action-based interaction rather than abstract explanation alone (44). If AI tools remain primarily text-driven, students with stronger kinesthetic preference scores may report lower perceived relevance of such tools. The adapted instruments were evaluated primarily using internal consistency measures (Cronbach's alpha), and additional psychometric validation (e.g., factor analysis, construct validity) was not conducted within the present study.

This finding may suggest the value of exploring AI integration strategies that connect digital guidance with physical execution in future instructional research. Approaches such as video-based feedback systems, motion capture analysis, and augmented reality may be relevant candidates for supporting kinesthetic learners, but their effectiveness was not tested in the present study (45). In this study, “learning profile concentration” refers to the degree of concentration of scores within one dominant VARK modality, rather than an inherent qualitative advantage. This pattern indicates greater concentration in reported VARK scores among students with higher self-reported AI use, but it should not be interpreted as evidence of increased consistency in learning strategies or reduced multimodal flexibility. Therefore, a more concentrated profile should not be interpreted as inherently advantageous from an educational perspective. The strong association between AI use and profile concentration (*β* = −.62) suggests greater concentration in reported VARK scores, but this should be interpreted cautiously.

Although motivation is central in PE and SDT offers a useful theoretical lens for discussing autonomy, competence, and relatedness (35, 36), the present study did not directly measure these constructs. Therefore, the findings should not be interpreted as evidence about motivation itself. At most, the observed patterns suggest that students who report greater AI use also report more favorable attitudes toward AI and stronger technology acceptance beliefs. Any interpretation regarding autonomy support, competence support, or broader motivational mechanisms remains theoretical and should be tested in future studies using validated SDT-based measures. One of the more exploratory findings is that AI usage frequency was associated with greater concentration in reported VARK score patterns. Lower dispersion values indicate a stronger concentration of preferences within a single modality, whereas higher values reflect more multimodal learning profiles; this distinction does not imply that either pattern is inherently advantageous. Students who reported more frequent AI use also reported greater concentration in particular VARK modalities, but this interpretation should remain cautious because the measure is derived from self-reported VARK scores in a cross-sectional design. One possible interpretation is that reported AI use co-occurs with preferences for written explanations or visual breakdowns, but the present data do not show whether AI use increased consistency in those preferences. Self-regulated learning theory may offer a useful interpretive framework, yet any mechanism linking AI engagement to learning preference patterns must be tested in longitudinal or experimental studies (37). This finding raises an important conceptual question. Future longitudinal research should examine whether these cross-sectional associations correspond to any changes in learning preference reports over time. Previous research suggests that over-structuring learning environments can reduce exploration and multimodal growth (38). Future research should test whether AI promotes balanced learning or modality fixation. The negative association between AI use and kinesthetic scores suggests a potential mismatch between predominantly text- or screen-based AI use and the movement-centered nature of PE, although causal or directional interpretations cannot be made. The findings may have cautious practical implications and tentative pedagogical relevance for PE contexts, but these implications should be interpreted cautiously because the present study did not measure learning outcomes, performance, or instructional effectiveness. First, future instructional research could examine how AI tools might be used as supplements rather than replacements for embodied practice. Technology may be most pedagogically appropriate when used to support feedback loops and reflection around movement tasks, although the present study did not test instructional effectiveness (39, 46). Second, educators may wish to consider whether AI-supported instruction is sufficiently inclusive of students with stronger kinesthetic preferences. In this respect, integrating AI outputs into active practice sessions may be a useful topic for future instructional research, but the present findings do not provide evidence that this approach improves learning, such as using AI-generated cues during drills or combining chatbot explanations with video modelling (32). Third, teacher training becomes essential. Research on AI literacy stresses that educators need skills to evaluate AI quality, guide ethical use, and prevent misuse or dependency (40). The results are consistent with the possible importance of teacher preparation for AI-supported instruction, but this implication should be treated as provisional rather than as a direct conclusion of the present study.

Students reported moderate safety perceptions. This is important because trust is associated with sustained adoption. Responsible AI frameworks emphasize transparency, data protection, and fairness as necessary conditions for educational implementation (41). PE contexts add further complexity because wearable data and performance monitoring raise privacy concerns. Ethical sport technology research highlights risks linked to biometric surveillance and unequal access. Institutions must therefore establish clear guidelines before expanding AI systems in PE curricula.

### Limitations and future research directions

This study has several limitations. First, the cross-sectional design precludes conclusions about causal direction; therefore, the reported relationships should be interpreted strictly as associations among self-reported variables. In addition, the conceptual model tested in this study was associative and relatively broad, so the findings should not be interpreted as confirming a fully specified causal or structural model among VARK patterns, technology acceptance beliefs, AI attitudes, and reported AI engagement. In particular, reverse or bidirectional relationships cannot be ruled out. For example, students with stronger visual or reading/writing preference patterns may be more likely to use current AI tools, which are largely text- and screen-based, just as more frequent AI use may be associated with those same reported preference patterns. The present design cannot determine temporal precedence between these variables. Second, AI-related perceptions was not directly measured using validated theoretical constructs such as autonomy, competence, and relatedness from Self-Determination Theory. As a result, the study cannot draw empirical conclusions about motivation, and any SDT-based interpretation remains theoretical. Third, all variables were self-reported, which may introduce common-method bias and response bias. Fourth, the instruments were adapted for the PE context, and psychometric evaluation in the present sample relied primarily on internal consistency rather than full construct validation. Psychometric evaluation of the PE-adapted instruments in the present sample relied primarily on internal consistency estimates. Confirmatory factor analysis, convergent or discriminant validity testing, and broader construct validation procedures were not conducted. Therefore, the adapted measures should be interpreted as contextually tailored instruments with preliminary reliability support rather than fully validated PE-specific scales. In addition, the use of VARK should be interpreted cautiously because learning-styles frameworks remain conceptually and empirically debated in contemporary educational research. Accordingly, VARK scores in the present study should not be interpreted as stable learner traits or as evidence for modality-matched instruction. Fifth, AI use was measured only through a single self-reported frequency indicator. Accordingly, the AI-use variable reflects reported or perceived academic engagement with generative AI rather than objectively verified behavior. It was not measured by type, purpose, duration, or quality of use. Sixth, the sample was drawn from a single academic context, which limits generalizability. Finally, the derived index of learning profile concentration should be considered exploratory and should not be interpreted as indicating better, more stable, or more valid reported learning preference patterns. Future work should also explore teacher perspectives, classroom climate effects, and the role of AI in practical skill acquisition rather than academic support alone. Research should also assess whether generative AI can support kinesthetic learners more effectively through multimodal interfaces, motion analysis, and immersive technologies (45).

The study did not measure instructional effectiveness, academic performance, skill acquisition, or learning outcomes. Therefore, the findings should not be interpreted as evidence for the effectiveness of specific pedagogical strategies or AI integration practices in physical education. The reported relationships should therefore be understood as associations observed at one point in time, rather than evidence that AI use changed students' VARK preference patterns or technology acceptance beliefs. In addition, the study did not objectively verify platform-specific activity, usage duration, prompt histories, interaction quality, or the extent to which AI outputs were integrated into PE-specific learning tasks. Future longitudinal studies should combine repeated VARK or learning-preference measurements with objective AI-use indicators, such as platform logs, AI-use diaries, time-on-task data, or teacher-verified AI-supported assignments, to examine whether these patterns change over time.

Importantly, reverse causality is a major interpretive limitation in the present study. The analyses were designed to test associations, not temporal order. Therefore, it is equally plausible that students with stronger visual or reading/writing preference patterns are more inclined to use existing AI tools because those tools are predominantly text- and screen-based. It is also possible that AI use and reported VARK patterns are reciprocally related, or that both are associated with broader factors such as study habits, digital confidence, academic orientation, or comfort with symbolic information. These possibilities cannot be disentangled in a single-time-point design.

Future research should include validated SDT-based instruments to directly assess autonomy, competence, and relatedness, in order to better understand motivational processes in AI-supported learning contexts. Future longitudinal and mixed-methods studies should verify AI use through more precise indicators, including platform-specific logs, usage duration, task type, prompt quality, and PE-specific learning outcomes.

### Contribution to the field

This study provides empirical evidence from a large sample of higher education students. It shows that self-reported generative AI use is associated with differences in reported learning preference patterns, attitudes toward AI, and technology acceptance beliefs in movement-based higher education. It extends technology acceptance research into physical education and highlights the need for pedagogically grounded AI integration strategies. Because the design is cross-sectional, the contribution lies in identifying associations rather than demonstrating effects. The findings are most applicable to higher education physical education contexts where students actively use digital tools. Generalization to other disciplines, age groups, or low-technology environments should be made with caution. Differences in instructional format and access to AI tools may influence the observed relationships.

## Conclusions

This study indicates that self-reported generative AI use was associated with several reported features of the learning environment in higher education physical education. Students reported moderately positive perceptions of AI usefulness and ease of use, and kinesthetic scores remained highest overall, consistent with the movement-based nature of the discipline. At the same time, more frequent self-reported AI use was associated with higher visual and reading/writing scores and lower kinesthetic scores. These findings should be interpreted as associative rather than causal, and they do not indicate changes in stable reported learning preference patterns, objectively verified AI use, or directly measured AI-related perceptions.

Technology acceptance beliefs were the strongest predictors of self-reported AI usage frequency, explaining a substantial proportion of variance in reported engagement. However, this variable reflects reported academic engagement with generative AI rather than objectively observed usage behavior. The findings should therefore be interpreted as associations among self-reported constructs, not as evidence about verified learning behavior or actual AI interaction patterns. Students with more visually and textually oriented reported preference patterns also tended to report stronger AI acceptance. In addition, higher self-reported AI use frequency was associated with greater concentration in reported VARK score patterns, although this finding should be treated as exploratory.

Overall, the findings support associations among self-reported AI use, attitudes toward AI, technology acceptance, and reported learning preference patterns.

The study does not establish causal effects, developmental change, or underlying mechanisms. In particular, it cannot determine whether reported AI use is associated with reported VARK patterns because AI use is greater among students with more visual or text-oriented preferences, because these reported preferences are associated with greater AI use, or because both are reciprocally related. The results are therefore best interpreted strictly as cross-sectional associations within a specific higher education physical education context.

## Data Availability

The raw data supporting the conclusions of this article will be made available by the authors, without undue reservation.
